# Membrane Phospholipid Fatty Acid Composition Regulates Cardiac SERCA Activity in a Hibernator, the Syrian Hamster (*Mesocricetus auratus*)

**DOI:** 10.1371/journal.pone.0063111

**Published:** 2013-05-01

**Authors:** Sylvain Giroud, Carla Frare, Arjen Strijkstra, Ate Boerema, Walter Arnold, Thomas Ruf

**Affiliations:** 1 Research Institute of Wildlife Ecology, Department of Integrative Biology and Evolution, University of Veterinary Medicine, Vienna, Austria; 2 University Medical Center Groningen, Departments of Chronobiology & Molecular Neurobiology, University of Groningen, Groningen, The Netherlands; 3 Department of Chronobiology, University of Groningen, Groningen, The Netherlands; 4 Wildlife Management, University of Applied Sciences Van Hall Larenstein, Leeuwarden, The Netherlands; 5 University Medical Center Groningen, Nuclear Medicine and Molecular Imaging, University of Groningen, Groningen, The Netherlands; Max Delbrueck Center for Molecular Medicine, Germany

## Abstract

Polyunsaturated fatty acids (PUFA) have strong effects on hibernation and daily torpor. Increased dietary uptake of PUFA of the n-6 class, particularly of Linoleic acid (LA, C18:2 n-6) lengthens torpor bout duration and enables animals to reach lower body temperatures (T_b_) and metabolic rates. As previously hypothesized, this well-known influence of PUFA may be mediated via effects of the membrane fatty acid composition on sarcoplasmic reticulum (SR) Ca^2+−^ATPase 2a (SERCA) in the heart of hibernators. We tested the hypotheses that high proportions of n-6 PUFA in general, or specifically high proportions of LA (C18:2 n-6) in SR phospholipids (PL) should be associated with increased cardiac SERCA activity, and should allow animals to reach lower minimum T_b_ in torpor. We measured activity of SERCA from hearts of hibernating and non-hibernating Syrian hamsters (*Mesocricetus auratus*) *in vitro* at 35°C. Further, we determined the PL fatty acid composition of the SR membrane of these hearts. We found that SERCA activity strongly increased as the proportion of LA in SR PL increased but was negatively affected by the content of Docosahexaenoic acid (DHA; C22:6 n-3). SR PL from hibernating hamsters were characterized by high proportions of LA and low proportions of DHA. As a result, SERCA activity was significantly higher during entrance into torpor and in torpor compared to inter-bout arousal. Also, animals with increased SERCA activity reached lower T_b_ during torpor. Interestingly, a subgroup of hamsters which never entered torpor but remained euthermic throughout winter displayed a phenotype similar to animals in summer. This was characterized by lower proportions of LA and increased proportions of DHA in SR membranes, which is apparently incompatible with torpor. We conclude that the PUFA composition of SR membranes affects cardiac function via modulating SERCA activity, and hence determines the minimum T_b_ tolerated by hibernators.

## Introduction

Torpor and hibernation are states with reduced metabolic rate and hence body temperatures (T_b_), a strategy employed by many birds and mammals to survive harsh environmental conditions [1]. However, vital organs must remain functional despite depressed oxygen consumption and single-digit T_b_. This is most important for the heart that has to maintain circulation by regular contractions to guarantee sufficient perfusion of the organism.

At T_b_ below 20°C, non-hibernators experience severe arrhythmias and ventricular fibrillation that leads to cardiac arrest [2]. This heart dysfunction is due to a massive increase in cytosolic calcium accompanied by calcium waves and by calcium overload [3], [4]. In contrast to non-hibernators, hibernating mammals remain in sinus rhythm even if T_b_ approaches 0°C [2]. This outstanding ability of the hibernator’s heart is due to the maintenance of sufficiently fast calcium removal into the sarcoplasmic reticulum (SR) after contraction, despite low T_b_ (see [5] and [4] for reviews). Indeed, an increased rate of calcium reuptake and larger calcium stores were reported in the SR of hibernating Richardson’s ground squirrels [6]. Moreover, the mRNA and protein levels of SR-calcium ATPase (SERCA 2a in the heart, subsequently called SERCA for simplicity), the pump removing calcium into the SR, were found to be increased by 3-fold in myocytes of hibernating woodchucks compared to those of animals in the non-hibernating season [7]. These results support the role of SERCA as the key enzyme that ensures proper calcium handling in cardiomyocytes and hence functioning of the heart at low T_b_.

Interestingly, the activity of this transmembrane pump seems to be affected by the fatty acid composition of the surrounding phospholipids (PL). Specifically, there are reports of positive effects of certain polyunsaturated fatty-acids (PUFA), namely those of the n-6 class, on SERCA activity in non-hibernating mice. Small increases in the n-6/n-3 PUFA ratio were associated with large increases in SERCA activity [8]. It was recently hypothesized that such an enhancing effect of n-6 PUFA on SERCA activity and therefore on calcium handling could explain the maintenance of proper cardiac function at low T_b_ [9]. This hypothesis could also explain data from *in-vivo* studies in hibernators. Both experimental trials and field studies showed positive effects of increased PUFA content in the diet, or in white adipose reserves, on torpor bout duration, tolerance of low T_b_, and energy savings [10]–[15]. Although not explicitly discriminated in these papers, the major PUFA added to the diet in these studies was Linoleic acid (LA, C18:2) of the n-6 family. Conversely, several studies reported negative effects of n-3 PUFA on SERCA activity [8], [16], [17] and it seems that n-3 fatty acids have adverse effects on hibernation [18]. To our knowledge, two studies [19], [20] have tested so far the effect of a diet specifically enriched in a n-3 fatty acid (α-Linolenic acid, LNA) on hibernation, but only one [19] assessed the fatty acid composition in the tissues. The authors found that a n-3 PUFA enriched diet, leading to a high content in adipose tissues, strongly reduced the propensity to hibernate in yellow bellied-marmots [19]. Taken together, these data suggest antagonistic effects of n-6 and n-3 PUFAs on SERCA activity, cardiac function and hence hibernation.

A role for membrane composition in regulating hibernation was further supported by the finding that free-living alpine marmots show a rapid increase of n-6 PUFA in PL just prior to hibernation, with highest levels reached in the heart [21]. Seasonal changes in the n-6 and n-3 PUFA contents of heart PL have also been reported in hibernators fed a constant diet. Specifically, higher levels of LA (C18:2 n-6) and Arachidonic acid (AA, C20:4 n-6) and lower proportions of Docosahexaenoic acid (DHA, C22:6 n-3) and Docosapentaenoic acid (DPA, C22:5 n-3) were reported in hibernating squirrels compared with summer active animals [22]. More recently, Geiser *et al.* [23] demonstrated that exposure to short photoperiod triggers a significant increase in n-6 PUFA and a concomitant reduction in n-3 PUFA (notably C22:6 n-3) in muscle PL of *Peromyscus maniculatus*, a species that exhibits daily torpor mainly under short days. Together, these findings point to an up-regulation of n-6 PUFA content and down-regulation of n-3 PUFA content in cardiac PL prior to hibernation or torpor that is independent of dietary intake. It seems that these changes in cardiac PL composition may be a prerequisite for animals to enter hibernation, possibly to keep the heart functioning at low T_b_.

In the present study, we therefore determined SERCA activity in hearts from hibernating Syrian hamsters (*Mesocricetus auratus*), and investigated its relation to the fatty acid composition of SR PL. Specifically, we tested the hypotheses that high proportions of n-6 PUFA in general, or specifically in LA (C18:2 n-6), relative to n-3 PUFA in SR phospholipids should be associated with increased cardiac SERCA activity, and should allow animals to reach lower minimum T_b_ in torpor.

## Materials and Methods

### Ethics Statement

The experiment was approved by the Animal Experimental Ethics Committee of the University of Groningen under license numbers DEC-2954 and DEC-4746a.

### Animals

Syrian hamsters (*Mesocricetus auratus*, 18 males, 29 females), five months old, were obtained from the breeding colony of the Zoological Laboratory (University of Groningen, Haren, The Netherlands) in two different years (2003 and 2008). Each year, animals were transferred to a climate-controlled room and were kept in standard laboratory cages under a summer photoperiodic regime (LD 14:10) at an ambient temperature of 21±1°C. Some hamsters were sacrificed under these conditions (summer, n = 7). After 9 weeks of acclimatization, the light-dark cycle was changed to a short photoperiod (LD 8:16) with a constant ambient temperature kept at 21°C, in order to induce a winter phenotype. After another 5–7 weeks, ambient temperature was changed to 5±1°C with continuous dim red light (<0.5 Lux), to trigger hibernation [24]. At all stages, the animals had *ad libitum* access to lab chows (Arie Blok Diervoeding, Woerden, The Netherland, www.arieblok.nl) and water. Hamsters were maintained under these constant conditions until sacrificed at different states within the hibernating cycle. All animals were killed by decapitation, 10 minutes after intra-peritoneal injection of pentobarbital (60 mg/Kg). T_b_ was measured immediately after decapitation in the animal’s mouth using a Voltcraft K102 (Conrad, the Netherlands) thermometer, equipped with a polytetrafluoroethylene insulated 1 mm bare tip copper constantan (Type T) thermocouple. Prior to use, the thermometer was calibrated in a water bath between 0°C and 45°C, by using a mercury thermometer (accuracy ±0.25°C). Hearts were quickly removed and stored at −80°C until subsequent determination of SERCA activity and SR PL composition at the Research Institute of Wildlife Ecology.

### Time-points in the Hibernating Cycle

Hibernation cycles were defined as a succession of torpid and euthermic states, lasting up to 7 days. Locomotor activity of hamsters was continuously monitored with passive infrared motion detectors. Animals were sacrificed during midwinter hibernation cycles of maximal length, i.e. between the 5^th^ and 10^th^ bout of torpor [24], at three different time-points: during entrance into torpor while T_b_ was still decreasing (“cooling”, n = 9), during deep torpor (“torpid”, i.e. at least 24 h after cessation of locomotor activity, n = 18) and during inter-bout arousal (“IBA”, n = 13). Samples during IBA were obtained from artificially aroused hamsters. These animals were re-warmed gently with both hands after at least three days of torpor and were sacrificed between 2.5 and 8.5 h after the onset of locomotor activity.

Some hamsters never entered hibernation and are termed “non-hibernating winter animals” (n = 7). The Syrian hamster is known as a facultative hibernator typically entering hibernation when challenged with low ambient temperatures and/or reduced food availability in addition to exposure to short photoperiod [25]. The availability of food throughout winter was presumably one of the reasons why 7 out of 47 experimental hamsters did not hibernate.

### Isolation of Cardiac SR

Cardiac homogenates were prepared from approximately half of each hamster heart (∼300 mg). Hearts were washed and minced in ice-cold isotonic saline (0.9% NaCl). A protease inhibitor cocktail (Sigma P8340) was added to the suspensions. Tissues were then homogenized in 2 ml of a buffer containing 30 mM histidine (pH 6.9), 300 mM sucrose, 600 mM KCl, 0.5 mM DTT, 10 mM EDTA, 50 mM Na_2_HPO_4_ and 1 mM PMSF, by 10 strokes with a motor-driven Teflon/glass homogenizer (tube volume, 5 ml, Wheathon, USA). Homogenates were then centrifuged at 24.600 g for 15 min to remove mitochondria, cell debris and most of the membranes, including sarcolemma, but not SR. Homogenates therefore contained SERCA protein embedded in membrane vesicles, and SERCA represents the major fraction (∼80%) of the total membrane protein of the longitudinal SR [26]. Supernatants with 10% glycerol were stored in 200 µl-aliquots at −80°C. All steps were performed at 4°C within 1 h to minimize enzyme denaturation.

### SERCA Activity Measurements

**Figure 1:**
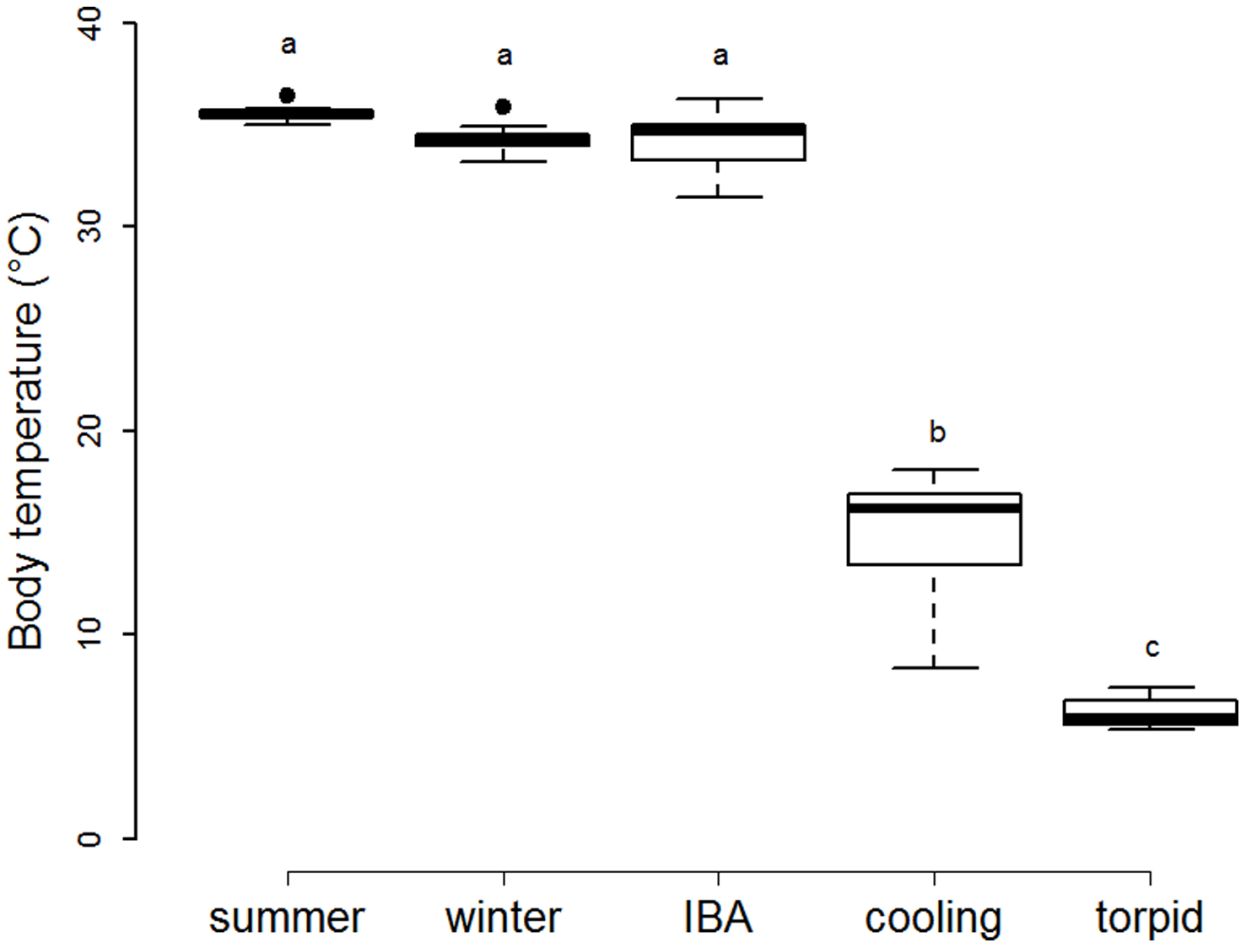
Body temperatures according to the animal physiological states. Body temperatures of summer hamsters (“summer”, n = 7), non-hibernating winter hamsters (“winter”, n = 7), and hibernating hamsters during inter-bout arousal (“IBA”, n = 13), entrance into a hibernating bout (“cooling”, n = 9), and deep torpor (“torpid”, n = 18). Groups differing significantly (p<0.001, Tukey’s post-hoc comparisons) are denoted by different superscripts.

ATPase activities were measured by a standard coupled enzyme assay, in which the rate of ATP hydrolysis was calculated from spectrophotometric recording (Perkin-Elmer 550, Germany) of NADH oxidation at 340 nm (ε = 6.22 mM^−1^cm^−1^). The assay was performed according to the method previously described by Simonides *et al.* [27], using a sample volume optimized for the Syrian hamster’s heart. SERCA activity was determined by adding 100 nM thapsigargin (TG), a specific inhibitor of SERCA [28], during the assay. Only SERCA protein embedded in the SR membrane vesicles remains functional. The difference between ATPase activities recorded before and after TG addition was attributed to SERCA. The standard reaction mixture contained 50 mM imidazole (pH 6.9), 100 mM KCl, 10 mM MgCl_2_, 10 mM NaN_3_, 0.5 mM DTT, 10 mM PEP, 5 mM ATP, 10 µM CaCl_2_, 5.3 unit.ml^−1^ pyruvate kinase, 17.5 unit.ml^−1^ lactate dehydrogenase, 300 µM NADH and 2 µM calcium ionophore (A23187) in a final volume of 1 ml. The reaction was started by addition of the sample (0.1–0.2 µg.µl^−1^ final volume). Assays were performed at a temperature of 35°C, which corresponds to euthermic T_b_ (Fig. 1). Protein concentrations were determined with the Bradford method [29]. Data presented are means from 4 replicate measurements of SERCA activities, and are expressed as µmol ATP hydrolyzed per mg total protein and minute.

### Lipid Analyses

Lipids were extracted from the isolated SR membranes following the procedure of Folch *et al.* [30]. The phospholipids (PL) were then isolated using thin-layer chromatography [31], and further saponified and converted into fatty acid methyl esters (FAME) as reported previously [32]. FAME were identified by gas-liquid chromatography using a Perkin-Elmer FID AutoSystem XL autosampler chromatograph equipped with a 30 m×60.25 mm×60.25 mm HP INNOWax capillary column, using the following parameters: injector 240°C, column 130–180°C at 4°C/min, 180–200°C at 3°C/min, 200–240°C at 15°C/min, 240°C for 8 min. The relative fatty acid composition was quantified using external fatty acid methyl ester standards (Supelco) run after every 20 samples and Turbochrom 6.3 software (Perkin Elmer). Concentrations of single fatty acids were calculated as mass % of total identified peaks of 13 fatty acids of chain length 14 to 22.

### Statistics

Data analyses were carried out using R 2.15.1 [33]. Residuals from statistical models were tested for normality (using Shapiro-Wilk tests), and if necessary, response variables were log-transformed. General linear models with Tukey-like post-hoc multiple comparison tests (R package ‘multcomp’ [34]) were used to assess differences in SERCA activity and SR PL composition between summer active animals, non-hibernating winter animals, and hibernating animals during IBA, cooling and torpor. For regression analyses we applied ranged major axis (RMA) models (R package ‘lmodel2’ [33]) to account for measurement errors in both the dependent and predictor variable. Finally, we carried out a path analysis (R package ‘lavaan’ [35]) to determine the most likely cause and effect relationship between the proportion of a given fatty acid among SR PL, SERCA activity and T_b_ of the animals. All reported values are means ± standard deviation.

## Results

### Body Temperature

T_b_ of hamsters was close to ambient during deep torpor, on average 1.7±0.6°C above, and significantly lower than in animals sacrificed during entrance into hibernation. T_b_ of summer hamsters was similar to the T_b_ of non-hibernating hamsters during winter and to the T_b_ of hamsters during IBA (Fig. 1).

### Cardiac SERCA Activity

**Figure 2:**
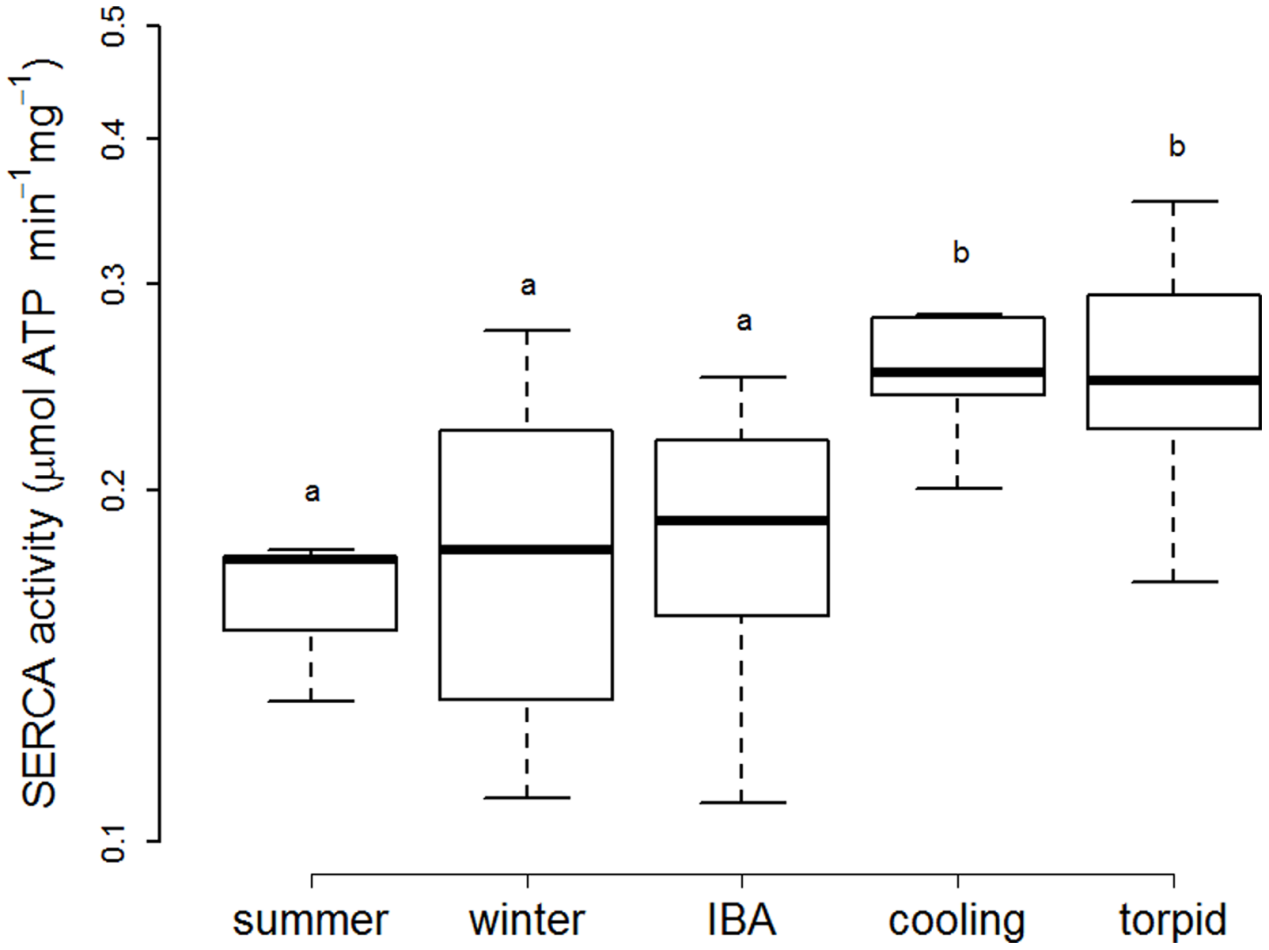
Levels of cardiac Sarcoplasmic Reticulum Calcium ATPase 2a (SERCA) activity. Levels of SERCA activity in summer hamsters (“summer”, n = 7), non-hibernating winter hamsters (“winter”, n = 7), and hibernating hamsters during inter-bout arousal (“IBA”, n = 13), entrance into a hibernation bout (“cooling”, n = 9), and deep torpor (“torpid”, n = 18). Groups differing significantly (p<0.01, Tukey’s post-hoc comparisons) are denoted by different superscripts.

**Figure 3:**
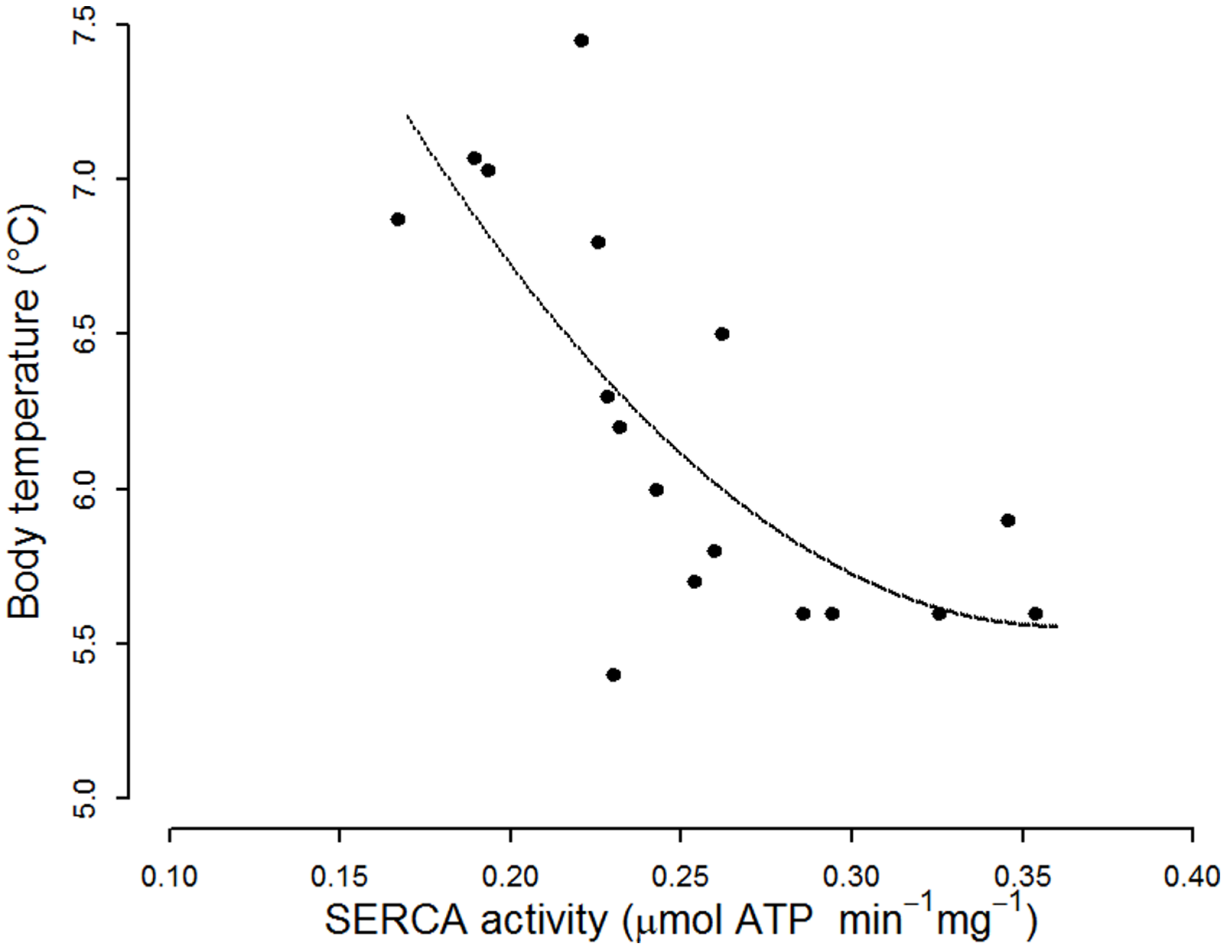
Body temperature as a function of cardiac Sarcoplasmic Reticulum Calcium ATPase 2a (SERCA) activity in torpid hamsters. Exponential ranged major axis regression: intercept = 8.99, slope = −10.78, p<0.01).

A general linear model revealed significant differences in SERCA activities in hearts from summer, non-hibernating winter, IBA, cooling and torpid hamsters (F = 9.42, p<0.001). SERCA activities, regardless of the original T_b_ of the hamsters, were all measured *in vitro* at 35°C. This resulted in the cancellation of Arrhenius effects in order to reveal the impacts of fatty acids on SERCA activity. SERCA from cooling hamsters had higher activity, similar to SERCA from torpid individuals, whereas SERCA from hamsters during IBA as well as from non-hibernating winter and summer animals had significantly lower activity (Fig. 2). Relating T_b_ of torpid animals, measured immediately before obtaining the tissue for SERCA preparation, to SERCA activity determined *in vitro* at 35°C, we found that minimum T_b_ reached in deep torpor decreased as SERCA activity increased (Fig. 3).

### Phospholipid Composition of Heart Sarcoplasmic Reticulum Membranes

In cardiac SR membrane PL, LA (C18:2 n-6), AA (C20:4 n-6) and DHA (C22:6 n-3) were the three major PUFA comprising 96.95% (±0.74%) of the total PUFA content. Proportions of LA were significantly lower (Table 1, F = 10.32, p = 0.002), and proportions of DHA significantly higher (Table 1, F = 8.81, p = 0.005), in hamsters in the non-hibernating state compared to animals in the hibernating state. This suggests that values from IBA hamsters rather resembled those of cooling and torpid animals (Table 1), despite a T_b_ similar to summer and non-hibernating winter animals (Fig. 1). Post-hoc tests between all five groups did not contradict these results, although not all single comparisons reached statistical significance. For instance, LA levels of non-hibernating winter hamsters were statistically equivalent to those of summer and IBA animals, which in turn did not significantly differ from those of cooling and torpid animals (Table 1). Regarding DHA, non-hibernating winter hamsters had significantly higher levels compared with any of the hibernating states (cooling, torpid, IBA), but DHA levels of summer animals did not significantly differ from those of all the other physiological states (Table 1). As to be expected, the LA/DHA ratio was significantly higher in hibernating hamsters *versus* non-hibernating animals (Table 1; F = 12.00, p = 0.001), as was the total n-6/n-3 PUFA ratio (Table 1; F = 7.43, p = 0.009), despite the lack of a corresponding difference in the second n-6 PUFA AA (Table 1; F = 0.001, p = 0.98). From the remaining n-3 PUFA only ALA (C18:3 n-3) differed significantly between the five compared groups due to, for some reason, a slightly higher concentration in cooling, and possibly also torpid animals (Table 1; comparison of hibernating state *vs.* non-hibernating state, F = 3.87, p = 0.055). However, concentrations of ALA in SR PL were in all groups very low (<0.5%, Table 1). The n-6/n-3 PUFA ratio in non-hibernating winter hamsters did not significantly differ from that of the summer and cooling animal groups, which had ratios similar to torpid and IBA hamsters (Table 1).

**Table 1:** Fatty acid proportions (% of total fatty acids) of cardiac sarcoplasmic reticulum phospholipids (means ± standard deviation), ratio of certain fatty acid proportions, and unsaturation index (“UI”) of summer hamsters (“Summer”, n = 7), non-hibernating winter hamsters (“Winter”, n = 7), and hibernating hamsters during entrance into a torpor bout (“Cooling”, n = 9), during deep torpor (“Torpid”, n = 18) and during inter-bout arousal (“IBA”, n = 13).

Fatty acid	Non-hibernating state		Hibernating state			ANOVA	
	Summer	Winter	Cooling	Torpid	IBA	F-statistic	p-value
C14:0	0.05±0.01^ab^	0.06±0.02^a^	0.05±0.01^ab^	0.04±0.01^b^	0.05±0.01^a^	7.30	**<0.001**
C15:0	0.14±0.04^a^	0.11±0.05^a^	0.06±0.01^b^	0.06±0.01^b^	0.07±0.01^b^	15.08	**<0.001**
C16:0	20.49±1.09^a^	18.60±2.51^ab^	16.54±1.51^bc^	16.70±1.28^c^	17.64±1.28^bc^	3.98	**<0.001**
C16:1 (n-7)	0.25±0.04^a^	0.27±0.08^ab^	0.35±0.07^abc^	0.36±0.08^bc^	0.39±0.08^c^	5.67	**<0.001**
C17:0	0.88±0.22^a^	0.65±0.23^b^	0.50±0.04^b^	0.51±0.07^b^	0.57±0.08^b^	12.44	**<0.001**
C18:0	19.92±0.94^a^	23.03±0.98^b^	23.31±0.78^b^	23.27±2.75^b^	22.72±0.97^b^	5.04	**0.002**
C18:1(n-9)	9.88±1.06^a^	8.38±0.83^b^	8.45±1.05^b^	8.41±0.85^b^	8.58±1.03^b^	3.45	**0.015**
C18:2 (n-6)	21.61±2.20^ab^	20.47±2.41^a^	24.18±1.07^b^	23.77±2.91^b^	22.53±2.35^ab^	3.58	**0.012**
C18:3 (n-3)	0.19±0.08^ab^	0.19±0.16^a^	0.37±0.04^bc^	0.28±0.15^ab^	0.19±0.15^a^	3.60	**0.012**
C20:4 (n-6)	15.69±1.61	15.82±1.90	15.09±1.24	15.56±2.27	16.43±2.09	0.70	0.598
C20:5 (n-3)	0.10±0.03	0.10±0.04	0.14±0.03	0.12±0.03	0.11±0.03	2.36	0.066
C22:5 (n-3)	1.07±0.19	1.00±0.41	1.26±0.13	1.22±0.21	1.13±0.30	1.59	0.191
C22:6 (n-3)	9.73±1.29^ab^	11.25±1.23^a^	9.51±1.10^b^	9.40±0.69^b^	9.50±1.32^b^	4.11	**0.006**
PUFA	48.39±1.62	48.82±3.14	50.56±1.84	50.32±3.24	49.90±1.97	1.16	0.342
MUFA	10.13±1.06^a^	8.65±0.87^ab^	8.80±1.11^ab^	8.77±0.89^b^	8.97±1.08^ab^	2.81	**0.035**
SFA	41.48±1.70	42.45±3.02	40.46±2.04	40.58±3.69	41.05±1.79	0.72	0.582
∑ n-6	37.30±2.04^ab^	36.29±3.26^a^	39.27±1.17^ab^	39.32±2.96^b^	38.96±1.72^ab^	2.77	**0.038**
∑ n-3	11.09±1.46	12.53±1.30	11.29±1.17	11.00±0.64	10.94±1.55	2.48	0.056
n-6/n-3	3.43±0.60^ab^	2.93±0.51^a^	3.51±0.35^ab^	3.58±0.29^b^	3.64±0.64^b^	2.97	**0.029**
C18:2/C22:6	2.26±0.40^ab^	1.85±0.39^a^	2.58±0.37^b^	2.55±0.40^b^	2.43±0.51^b^	4.09	**0.006**
UI	1.81±0.05	1.86±0.09	1.83±0.10	1.82±0.12	1.84±0.09	0.32	0.865

The unsaturation index was calculated as followed: UI = Σ [(% each fatty acid) (no. double bonds/fatty acid)]. Groups differing significantly with p<0.05 (Tukey’s post-hoc comparisons) are denoted by different superscripts.

We also found significant differences among several proportions of saturated fatty acids, such as Palmitic acid (C16:0), Stearic acid (C18:0) between the five groups (Table 1). These effects were, however, due to differences between summer and winter animals but unrelated to thermoregulatory states (Table 1). The same result was found for monounsaturated fatty acids.

### Cardiac SERCA Activity and Phospholipid Composition

**Figure 4:**
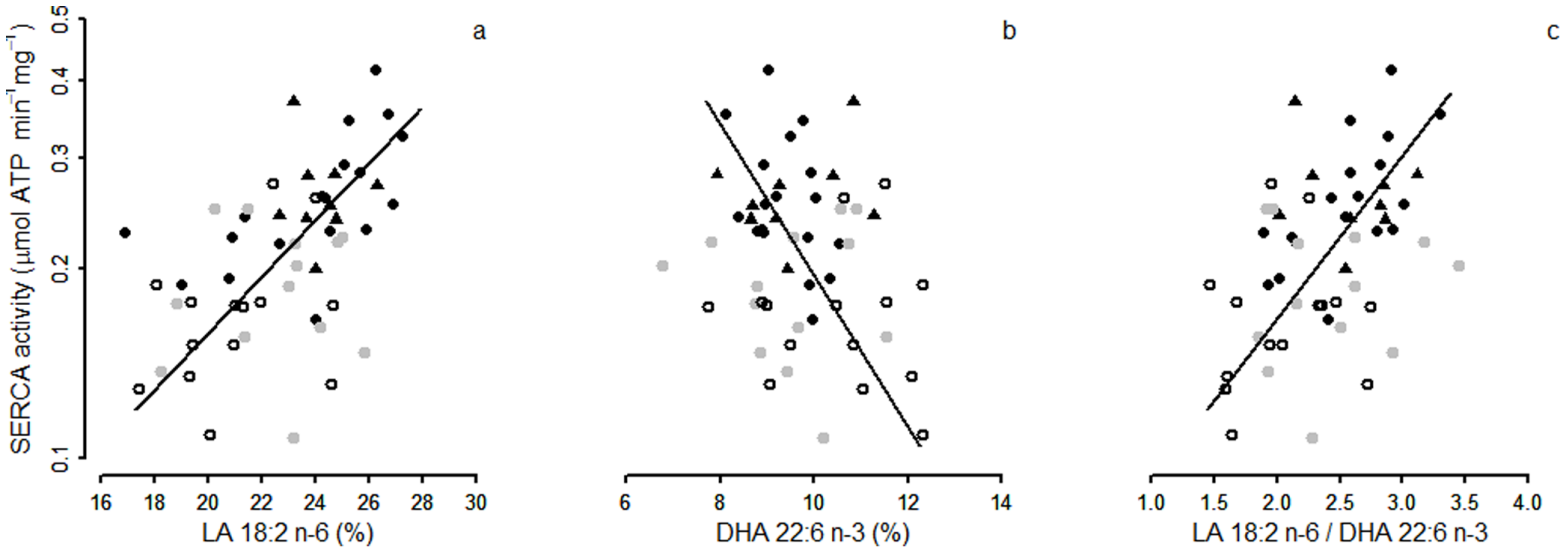
Cardiac Sarcoplasmic Reticulum Calcium ATPase 2a (SERCA) activity and fatty acid composition. SERCA activity as a function of the proportions (% of total fatty acids) of (a) Linoleic acid (LA 18:2 n-6), (b) Docosahexanoic acid (DHA 22:6 n-3), and (c) the ratio of LA/DHA in the sarcoplasmic reticulum membrane. Black dots indicate data from torpid animals, black triangles from cooling animals, grey dots from animals during inter-bout arousals, and open dots from summer and non-hibernating winter animals (Difference of slopes between groups: (a) F = 1.78, p = 0.15; (b) F = 0.89, p = 0.48; (c) F = 2.07, p = 0.10; regression statistics for all 54 animals from linear ranged major axis analysis: (a) intercept = −1.70, slope = 0.05, adjusted R^2^ = 0.27, p<0.01; (b) intercept = 0.50, slope = −0.12, adjusted R^2^ = 0.04, p = 0.04; (c) intercept = −1.29, slope = 0.03, adjusted R^2^ = 0.18, p = 0.02).

SERCA activity was positively associated with the proportion of LA among SR PL (Fig. 4a), but negatively with the proportion of DHA (Fig. 4b), and thus positively with the ratio of LA/DHA (Fig. 4c). These relations were also significant for torpid animals only (LA: intercept = −1.39, slope = 0.03, adjusted R^2^ = 0.33, p = 0.01; DHA: intercept = 0.65, slope = −0.13, adjusted R^2^ = 0.12, p<0.05; LA/DHA: intercept = −1.21, slope = 0.24, adjusted R^2^ = 0.37, p = 0.01), i.e., for the state when the presumed influence of PL-composition on SERCA activity is most critical. No significant relation was found between SERCA activity and the n-6/n-3 PUFA ratio (intercept = −2.92, slope = 0.64, p = 0.15). SERCA activity increased, however, with the proportion of DPA (C22:5 n-3), the precursor of DHA, both among all hamsters and in torpid animals only (intercept = −1.22, slope = 0.47, adjusted R^2^ = 0.18, p = 0.01), but the proportion of DPA among SR PL was only <2%. Also, no significant association existed between AA and SERCA activity.

### Causal Relations

**Figure 5:**
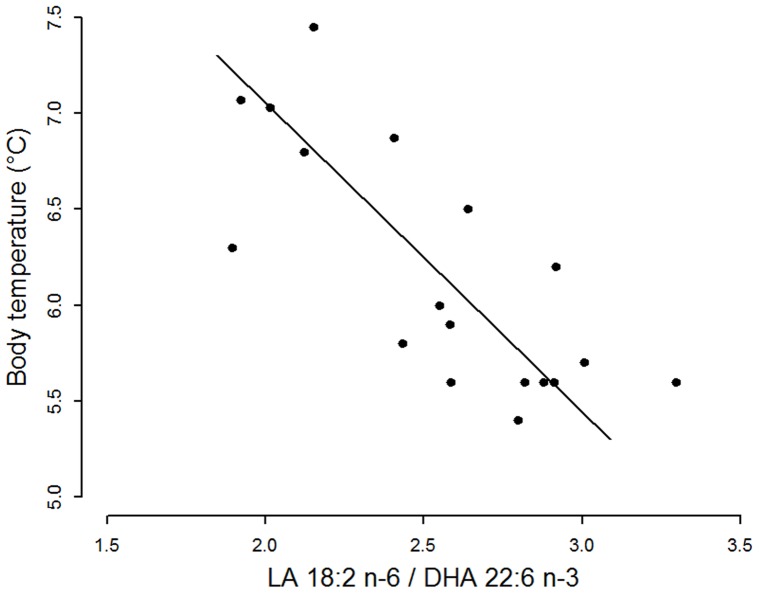
Body temperature as a function of the ratio of the proportions (% of total fatty acids) of Linoleic acid and Docosahexanoic acid (LA 18:2 n-6/DHA 22:6 n-3) in the sarcoplasmic reticulum membrane of torpid hamsters. Linear ranged major axis analysis: intercept = 10.29, slope = −1.61, p<0.01, R^2^ = 0.40.

Path analysis indicated that the proportion of LA among SR PL, and, to a lesser degree that of DHA, were associated with SERCA activities, which in turn determined the minimum T_b_ reached (Table 2). Results of this causal analysis were significant both in the pooled sample from all states discriminated but also for torpid animals only. As a consequence, minimum T_b_ in torpid animals decreased as the LA/DHA ratio increased (Fig. 5).

**Table 2:** Path analyses between Linoleic acid (LA) or Docosahexanoic acid (DHA), log-transformed cardiac Sarcoplasmic Reticulum Calcium ATPase (SERCA2a) activity and body temperature (Tb) in all hamsters (summer, winter, cooling, torpid and inter-bout arousal) and for torpid animals only.

	All hamsters			Torpid hamsters		
Variables	Estimate	Z-value	p-value	Estimate	Z-value	p-value
*T_b_ ∼ SERCA2a*	−64.22±10.62	−6.05	**<0.001**	−4.44±1.02	−4.34	**<0.001**
*SERCA2a ∼ LA 18:2n-6*	0.03±0.01	4.62	**<0.001**	0.02±0.01	3.27	**0.001**
*SERCA2a ∼ DHA 22:6n-3*	−0.03±0.01	−1.88	0.06	−0.06±0.03	−1.93	**0.05**

The model detects potential causal effects between LA or DHA and SERCA activity (SERCA2a ∼ LA 18:2n-6, SERCA2a ∼ DHA 22:6n-3), and between SERCA activity and Tb (Tb ∼ SERCA2a) of the hamsters. The “Estimates” correspond to the coefficients of the different regressions calculated by the model, and inform on the relative strength of the respective dyadic relations.

## Discussion

### Up-regulated SERCA Activity in Membranes Rich in LA

Our results suggest that a high proportion of LA in the SR membrane, rather than a high total n-6 PUFA content, up-regulates SERCA activity and determines the minimum T_b_ a hibernator can tolerate during torpor. This conclusion is mainly based on the significant correlation between SERCA activity and levels of LA (Fig. 4a). SERCA activity was therefore not affected by the n-6/n-3 PUFA ratio, as previously suggested [9]. Indeed, the effect in the Syrian hamster was specific to LA, as we found no association between AA and SERCA activity. T_b_ of torpid hamsters measured in this study can be considered as the minimum T_b_ reached during the respective torpor bout. Syrian hamsters reach minimum T_b_ under ambient temperatures like in this experiment within 24 hours, thus long before samples were taken from torpid animals [36], [37]. Therefore, the observed negative association between T_b_ of torpid animals and SERCA activity would suggest that incorporation of LA into the sarcoplasmic membrane may compensate Arrhenius effects on SERCA activity and thus enable tolerance of lower T_b_. Lastly, our path analysis supports such a sequence of causal relations.

Previous studies are in line with this major result of our study. Hibernating Richardson’s ground squirrels, for instance, show increased rates of calcium reuptake and larger SR calcium stores compared to active animals [6], [38]. However, these earlier studies failed to demonstrate significantly higher SERCA activity during hibernation, although this pump removes 70–92% of the total calcium present in the cytosol after excitation and contraction of cardiac myocytes [39]. Furthermore, an enhanced cytosolic clearance of calcium has been observed in hibernating ground squirrels *versus* non-hibernating rats, and this difference was attributed to differences in SERCA activity [3].

SERCA protein levels have been reported to be increased by 3-fold in myocytes of hibernating woodchucks compared to those of animals in the non-hibernating season [7]. In our study, total SERCA activity may have also been affected by changes in the amount of SERCA protein, i.e. between seasons. However, since our analysis shows effects of membrane fatty acid composition on SERCA activity per mg protein (e.g. Fig. 4), we would conclude that fatty acids affect the specific activity of the SERCA pump rather than the expression of SERCA protein. This relies on previous findings showing that SERCA accounts for the largest fraction of the total SR membrane protein [26]. Still further studies on this subject should validate this conclusion by additionally determining levels of either mRNA or protein levels of both SERCA and its inhibitor, phospholamban.

### Adverse Effects of Docosahexanoic Acid on SERCA Activity

In contrast to LA, SERCA activity was negatively associated with the DHA content of the SR membrane, although the relation was weak. Interestingly, the non-hibernating hamsters were characterized by high DHA but low LA content in their SR PL. Apparently, this phenotype is incompatible with the use of torpor. Hill and Florant [19] reported similar inhibitory effects of an ALA (the precursor of DHA) enriched diet on hibernation propensity in yellow bellied marmots. Among eight marmots tested, only three entered hibernation, whereas the other five individuals stayed euthermic throughout winter and continued to feed. Further, recent experiments conducted on garden dormice (*Eliomys quercinus*) revealed that animals fed a diet supplemented with fish oil, containing high amounts of DHA, significantly delayed the onset of hibernation compared to individuals fed a diet supplemented with corn oil high in LA (Giroud, Arnold and Ruf; unpublished data).

The apparent incompatibility of high DHA contents in PL with hibernation could be due the known strong inhibitory effects of n-3 fatty acids on SERCA activity [8], [16]. Such inhibition can be cardio-protective when operating at high T_b_, and is apparently mediated by DHA [40], [41]. Low T_b_, on the other hand, apparently requires removal of DHA and increased LA content in SR membranes leading to enhanced calcium handling in order to prevent cardiac arrest caused by Arrhenius effects [17].

### Potential Mechanisms

Although the actual biochemical mechanisms by which PUFA could influence SERCA activity are unknown, the most likely explanation would be that physical properties of certain unsaturated fatty acids affect the conformational change and hence the specific activity of the trans-membrane SERCA pump. These interactions are now well understood and can be described in terms of protein-induced perturbations to the membrane shape that feed-back on trans-membrane proteins (review in [42]). They may also involve specific chemical interactions. For example, biochemical modeling has recently demonstrated that certain fatty acids (DHA in the study) may displace interactions between protein domains and therefore facilitate the formation of active states [43]. One of the first membrane proteins that was shown to be affected by the properties of the surrounding lipids was in fact SERCA (review in [44]). Specifically, SERCA 1a activity in skeletal muscles was regulated by membrane contents of both cholesterol and phosphaditylethanolamine [44]. Our data suggest that, in the context of hibernation, the beneficial effects on cardiac SERCA are due to a replacement of DHA with LA in SR PL. Therefore, a previously suspected influence of the n-6/n-3 PUFA ratio [9] may only be correlational, caused by a potential inhibitory effect of DHA and a possible facilitating effect of LA on SERCA.

If a low content of DHA in SR PL is detrimental at high T_b_, but a high LA content necessary for adequate calcium handling during deep torpor, one would expect according membrane adjustments during rewarming and entrance into torpor. We did not find such differences between the SR membranes of IBA and cooling or torpid hamsters. This could be due to the fact that in our study IBA hamsters were sampled after artificially induced rewarming. This may have limited the time for the animals to complete changes in membrane composition, and explain the lack of significant alterations.Also, SERCA activity of IBA hamsters was similar to summer and non-hibernating winter animals (Fig. 2), despite a membrane composition that was intermediate between non-hibernating animals and cooling or torpid individuals (Table 1). This suggests additional influences on SERCA activity apart from those of membrane composition. A prime candidate molecule for such an influence is the SERCA inhibitor phospholamban (PLB) [45]. Generally, PLB gene expression has been found to be low during hibernation [7], [46], presumably because inhibition of SERCA is superfluous at low T_b_. However, even low levels of active PLB could be responsible for the unexpected low SERCA activity of our IBA hamsters. If so, PLB should be deactivated prior to entrance into the next torpor bout in order to guarantee maximal SERCA activity when Arrhenius effects become severe due to low T_b_. SERCA inhibition by PLB is relieved by phosphorylation via cAMP-dependent protein kinase, which in turn is activated through epinephrine release after sympathetic stimulation [47]. Interestingly, a sympathetic burst is typical prior to entry into torpor as described in some hibernating species [48], [49], and more recently in hibernating dormice [50]. Furthermore, it has been demonstrated experimentally that a functional sympathetic nervous system is a prerequisite for the use of torpor. Djungarian hamsters did not enter torpor after blockade of sympathetic action by administering 6-hydroxydopamine [51].

The immediate result of sympathetic stimulation is a transient increase of metabolic and heart rate, hence the opposite of what one would expect in preparation for torpor, but a phenomenon that has long been known to occur [52]. However, these inevitable side effects of sympathetic stimulation are short term, presumably in contrast to the effect on PLB. On one hand, Arrhenius effects could preserve phosphorylation and thus inactivation of PLB at low T_b_. On the other hand, repeated beta-adrenergic stimulation occurs during torpor when non-shivering thermogenesis is initiated in order to defend an already low T_b_ against a further decrease [48], [49], or for rewarming at the end of a torpor bout. In the latter case, efficient suppression of PLB may well be pivotal because heart rate is increasing tremendously although T_b_ is still very low [53]–[55].

### Conclusion and Perspectives

In conclusion, our findings suggest that replacing DHA with LA in SR PL up-regulates SERCA activity, thus possibly enabling hibernators to tolerate low T_b_ without jeopardizing heart function. In contrast, a high content of DHA in SR PL could be responsible for dampening SERCA activity to ensure proper calcium handling at high T_b_ [40], [41], but appears to be incompatible with the use of torpor. We are well aware that these findings correspond to associations between SR composition, SERCA activity and T_b_, which ask for more experimental follow-up studies. However, the fact that these associations can be detected even in animals fed identical rodent chow leads us to think that effects of differences in SR PL may be even stronger in free-living hibernators exposed to a higher variability in food resources, or even with limited access to essential PUFA [21]. Furthermore, the role of PLB during hibernation and its regulation by ß-adrenergic stimulation also needs experimental clarification by measuring levels of phosphorylated and dephosphorylated PLB during IBA, cooling, deep torpor, and rewarming.
